# Mutual Control of Cholinergic and Low-Threshold Spike Interneurons in the Striatum

**DOI:** 10.3389/fncel.2016.00111

**Published:** 2016-04-29

**Authors:** Rasha Elghaba, Nicolas Vautrelle, Enrico Bracci

**Affiliations:** Department of Psychology, The University of SheffieldSheffield, UK

**Keywords:** striatum, interneuron, acetylcholine, nitric oxide, GABA, mutual excitation

## Abstract

The striatum is the largest nucleus of the basal ganglia and is crucially involved in action selection and reward processing. Cortical and thalamic inputs to the striatum are processed by local networks in which several classes of interneurons play an important, but still poorly understood role. Here we investigated the interactions between cholinergic and low-threshold spike (LTS) interneurons. LTS interneurons were hyperpolarized by co-application of muscarinic and nicotinic receptor antagonists (atropine and mecamylamine, respectively). Mecamylamine alone also caused hyperpolarizations, while atropine alone caused depolarizations and increased firing. LTS interneurons were also under control of tonic GABA, as application of the GABA_A_ receptor antagonist picrotoxin caused depolarizations and increased firing. Frequency of spontaneous GABAergic events in LTS interneurons was increased by co-application of atropine and mecamylamine or by atropine alone, but reduced by mecamylamine alone. In the presence of picrotoxin and tetrodotoxin (TTX), atropine and mecamylamine depolarized the LTS interneurons. We concluded that part of the excitatory effects of tonic acetylcholine (ACh) on LTS interneurons were due to cholinergic modulation of tonic GABA. We then studied the influence of LTS interneurons on cholinergic interneurons. Application of antagonists of somatostatin or neuropeptide Y (NPY) receptors or of an inhibitor of nitric oxide synthase (L-NAME) did not cause detectable effects in cholinergic interneurons. However, prolonged synchronized depolarizations of LTS interneurons (elicited with optogenetics tools) caused slow-onset depolarizations in cholinergic interneurons, which were often accompanied by strong action potential firing and were fully abolished by L-NAME. Thus, a mutual excitatory influence exists between LTS and cholinergic interneurons in the striatum, providing an opportunity for sustained activation of the two cell types. This activation may endow the striatal microcircuits with the ability to enter a high ACh/high nitric oxide regime when adequately triggered by external excitatory stimuli to these interneurons.

## Introduction

The striatum is the largest nucleus of the basal ganglia and the main recipient of cortical and thalamic inputs (Zheng and Wilson, 2002; Gerfen and Surmeier, 2011). These excitatory inputs are locally processed and transformed into striatal outputs, which are crucial for motor control and action selection (Balleine et al., 2007; Pennartz et al., 2009). A complex striatal microcircuit, that includes several types of interneurons, is involved in this operation (Tepper and Bolam, 2004). While most GABAergic interneurons and all projection neurons of the striatum are not autonomously active, two well characterized interneuronal types, the cholinergic interneurons and the nitric oxide-expressing cells known as low-threshold spike (LTS) interneurons, generate spikes even in the absence of synaptic inputs (Bennett et al., 2000; Beatty et al., 2012). The continuous activity of these interneurons is expected to cause tonic release of neurotransmitters, which in turn is likely to contribute to the resting state of the striatal networks. *In vivo* experiments have shown that striatal tonically active neurons (TANS) also produce phasic responses, particularly to reward-related events (Apicella, 2007). TANS exhibit a characteristic pause in their firing after delivery of an unexpected reward, that coincides, at least partially, with the burst of action potentials generated by dopaminergic neurons in response to reinforcing stimuli (Joshua et al., 2008). While TANS have been historically identified as cholinergic interneurons, it has subsequently emerged that LTS interneurons have spontaneous firing patterns that are virtually indistinguishable from those of cholinergic interneurons (Beatty et al., 2012). Therefore it is highly likely that a part of the TANS recorded *in vivo* were LTS, rather than cholinergic, interneurons.

While the ability of the local GABAergic network to provide feedforward and feedback inhibition to projection neurons has been investigated in great detail (Koos et al., 2004), only more recently has the function of other interneurons started to emerge. Several studies have investigated the role of cholinergic interneurons in the striatal microcircuits (English et al., 2011) These cells control glutamatergic inputs to striatal projection neurons (Pakhotin and Bracci, 2007) and in doing so can gate corticostriatal inputs in response to thalamic activation (Ding et al., 2010). Furthermore, cholinergic interneurons are crucially involved in the control of dopamine release, being able to trigger such release even in the absence of action potentials in dopaminergic fibers (Threlfell et al., 2012). In comparison, the role of LTS interneurons is far less understood. In addition to nitric oxide synthase, LTS interneurons also express GABA, somatostatin and neuropeptide Y (NPY; Ibáñez-Sandoval et al., 2011). While the release of GABA by LTS interneurons can inhibit striatal projection neurons (Tepper et al., 2008), the influence of their other neurotransmitters is still poorly documented. Nitric oxide appears to control glutamatergic inputs to striatal projection neurons in a complex manner. In particular, this neurotransmitter has been shown to affect both short- and long-term plasticity of corticostriatal synapses (Centonze et al., 1999; Picconi et al., 2011; West and Tseng, 2011).

While cortical and thalamic glutamatergic inputs control the activity of both cholinergic and LTS interneurons (Partridge et al., 2009; Doig et al., 2014), previous evidence also raised the possibility that reciprocal interactions between these interneurons may exist. Excitation of cholinergic interneurons has been described in response to exogenous application of nitric oxide donors (Centonze et al., 2001) raising the possibility that release of endogenous nitric oxide by LTS interneurons could also exert control over these cells. Furthermore, activation of nicotinic and muscarinic receptors modulates tonic GABA levels in the striatum (Luo et al., 2013). Although it is not clear how this modulation may affect LTS interneurons, this finding suggests that acetylcholine (ACh) could exert both direct and indirect actions on these cells. Studies of LTS interneurons have been limited by their relative rarity and absence of morphological distinctive features. In order to cast light on these issues, we studied the interactions between LTS and cholinergic interneurons using two strains of transgenic animals in which LTS interneurons can be visually identified (Ibáñez-Sandoval et al., 2011) and photostimulated (Rafalovich et al., 2015).

## Materials and Methods

### Animals

All experiments were carried out in accordance with the 1986 Animal (Scientific Procedures) Act, and with approval from the UK Home Office. Recordings were obtained from two transgenic mouse lines. The first line comprised heterozygous BAC NPY-GFP mice in which a humanized Renilla green fluorescent protein (hrGFP, Stratagene) sequence was inserted into the start of the transitional site of the NPY gene (Stock 006417, Jackson Laboratory, USA). On these mice, NPY-expressing neurons also express GFP.

The second transgenic line (ChR2-SOM) was obtained by crossing homozygous SOM-IRES-Cre mice (Stock 013044, Jackson Laboratory, USA) with homozygous ChR2 (H134R)-eYFP mice (Stock 024109, Jackson Laboratory, ME, USA). The resulting offspring selectively express a channelrhodopsin2-yellow fluorescent protein (ChR2 (H134R)-eYFP) fusion protein in somatostatin-expressing neurons.

### Slice Preparation

Mice aged 25 ± 5 days were anesthetized with inhaled isoflurane and perfused transcardially with 2.5–5 ml of oxygenated ice cold modified HEPES artificial CSF (aCSF) solution containing (in mM): 92 NaCl, 2.5 KCl, 1.2 NaH_2_PO_4_, 30 NaHCO_3_, 20 HEPES, 25 Glucose, 5 sodium ascorbate, 2 Thiourea, 3 sodium pyruvate, 10 MgSO_4_.7H_2_O, 0.5 CaCl_2_.2H_2_O at pH 7.4. The animals were subsequently killed by decapitation and the brain was quickly removed from the skull. Using a vibroslicer (Camden instruments), parasagittal brain slices (250 μm thick) were prepared. Slices were immediately transferred to a recovery chamber containing modified HEPES aCSF continuously bubbled with a carbogen mixture of 95% O_2_ and 5% CO_2_ gas, were they were kept at 32°C for 15 min. Slices were then transfer red to a storage chamber which contained oxygenated standard aCSF at 25°C. The slices were left for at least 1 h to equilibrate before electrophysiological recordings.

For recordings, slices were transferred to a recording chamber and continuously superfused with oxygenated standard aCSF containing (in mM): 124 NaCl, 2.5 KCl, 1.2 NaH_2_PO_4_, 24 NaHCO_3_, 5 HEPES, 12.5 Glucose, 2 MgSO_4_.7H_2_O, 2 CaCl_2_.2H_2_O at pH 7.4 (flow rate 1.5 −2 ml/min) at 24–25°C.

### Slice Visualization and Optogenetic Stimulation

Slices were visualized using an infrared/differential interface contrast microscopy with a 40× water-immersion objective. For visualization of GFP- or eYFP-expressing neurons, we used epifluorescence with standard GFP filters; GFP or eYFP excitation was provided through either a mercury lamp (Olympus U-RFL-T) or a high power blue light LED driver (DC2100, ThorLabs). For photostimulation of ChR2 expressing neurons, we used a high power blue light LED driver (DC2100, ThorLabs) connected to the fluorescent port of an Olympus BX51 microscope. The size of the photostimulation spot was reduced to approximately 1–2 mm using the diaphragms of the fluorescence port.

### Electrophysiological Recording

For whole-cell recordings, patch pipettes (3–6 MΩ) were prepared by pulling borosilicate glass tubes with a PC-10 puller (Narishige). Pipettes were filled with an intracellular solution containing in (mM): 120 K-gluconate, 20 KCl, 2 MgCl_2_, 12 HEPES, 0.4 Na-GTP and 4 Na_2_-ATP, 0.04 EGTA, adjusted to pH 7.3 with KOH. Current-clamp recordings were performed in bridge mode using an NPI BA-1S bridge amplifier. For experiments in which spontaneous and evoked GABAergic inhibitory postsynaptic potentials (IPSPs) were studied, a high-chloride intracellular solution (in which equimolar KCl replaced K-Gluconate) was used to increase chloride driving force and therefore GABAergic responses.

**Figure 1:**
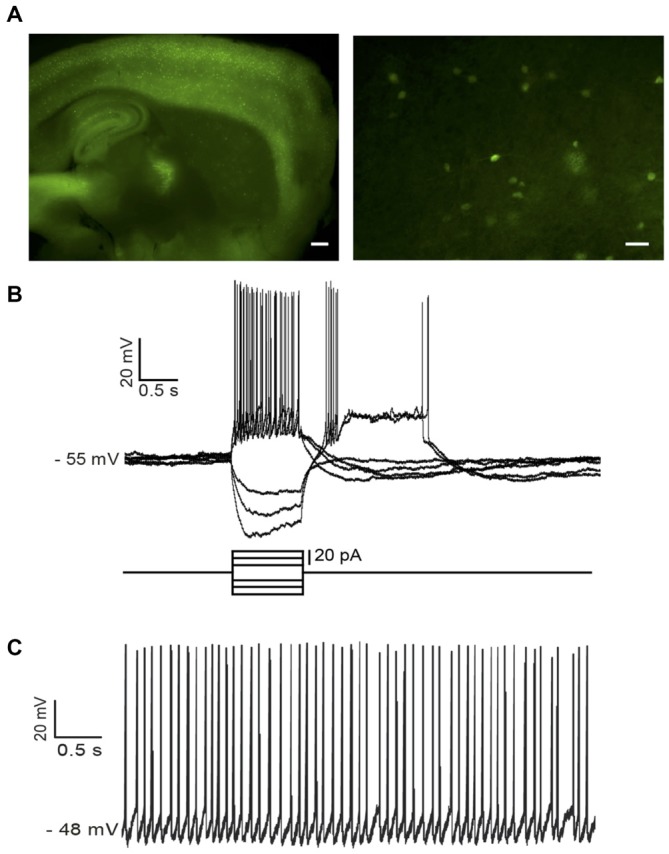
**Identification of low-threshold spike (LTS) interneurons in neuropeptide Y (NPY)-green fluorescent protein (GFP) mice. (A)** Fluorescent photomicrography of a typical parasagittal brain slice from an NPY-GFP mouse. GFP-positive neurons are visible both in the cortex and the hippocampus (at higher density) and in the striatum. The right-hand panel shows GFP-positive neurons in the striatum. These are mainly medium-sized cells, with a number of primary dendrites emerging from the soma (inset). Calibration bars: 500 μm (left panel), 200 μm (right panel). **(B)** Typical properties of a LTS interneuron as revealed by current injection steps, which were superimposed on a small steady current injection (−5 pA) delivered to abolish spontaneous spikes before current steps. Negative current steps were followed by prolonged rebound plateau potentials, accompanied by spikes. **(C)** An example of regular firing observed in a spontaneously active LTS interneuron. Properties similar to the ones of **(B)** could be elicited in all spontaneously active neurons if their firing was abolished by a steady negative current injection.

In NPY-GFP mice, NPY-expressing neurons also expressed GFP. In the striatum, these GFP-positive cells belong to two distinct interneuronal types: LTS interneurons and neurogliaform interneurons (NGFIs; Ibáñez-Sandoval et al., 2011; Logie et al., 2013). A parasagittal brain slice from an NPY-GFP mouse imaged with fluorescence microscopy is shown in Figure 1A (left). A larger magnification image of the striatum is shown in the right-hand panel. NPY-positive LTS interneurons were initially identified based on their fluorescence, usually slightly fainter than that of NGFIs (Ibáñez-Sandoval et al., 2011); once a whole-cell configuration was established, identification of LTS interneurons was confirmed based on their distinctive electrophysiological properties, that are revealed by current injections and include high (>0.5 GΩ) input resistance and prolonged rebound calcium spikes (Kawaguchi, 1993; Beatty et al., 2012; Cains et al., 2012), as shown in the example of Figure 1B. Most LTS interneurons were spontaneously active (Beatty et al., 2012) as shown in the example of Figure 1C.

In ChR2-SOM mice, eYFP and ChR2 are selectively expressed by somatostatin-expressing neurons that, in the striatum, correspond to LTS interneurons (Ibáñez-Sandoval et al., 2011). The electrophysiological properties of these neurons were checked at the beginning of each whole-cell recording from eYFP-positive neurons using current pulses (Figure 7B).

During IR microscopic slice observation, cholinergic interneurons were identified based on their very large somatic size. After establishment of a whole-cell configuration, their identity was confirmed upon observation of their distinctive electrophysiological properties probed with current pulses (Kawaguchi, 1993; Wilson, 2005; Blomeley et al., 2011). Cell-attached recordings were carried out using glass pipettes similar to those used for whole-cell recordings, filled with either ACSF or intracellular solution. In that configuration, cholinergic interneurons were identified based on their large somata, lack of fluorescence and presence of spontaneous extracellular firing.

Medium-sized neurons were classified as projection neurons (MSNs) based on their distinctive membrane properties in response to injections of negative and positive current, as in previous studies (Bracci et al., 2003; Blomeley et al., 2009; Blomeley and Bracci, 2011).

Spontaneous and evoked GABAergic IPSPs were recorded in the presence of the NMDA receptor antagonist D-AP5 (10 μM) and the AMPA receptor antagonist NBQX (10 μM), in order to block ionotropic glutamate receptors. Electrical stimulations (100–1000 μA, 100–1000 μs) were applied with a monopolar glass electrode (<0.1 MΩ) filled with ACSF and placed near the recorded cell in order to stimulate the local GABAergic fibers. Paired pulse stimulation (200 ms interval) was delivered continuously every 10 s in control solution and during application of pharmacological treatments. Data collected during the initial washing of the drugs (first 5 min) were excluded from analysis. At least 30 paired-pulse responses were present in each pharmacological condition considered in this study. For analysis of the amplitude of the first response, in each experiment, the amplitudes of the responses evoked by the first stimulus were normalized to the average amplitude of these responses in control solution. Paired-pulse ratios (PPR) were calculated as the ratio between the amplitudes of second evoked IPSP and the first evoked IPSP. For each experiment, the amplitudes of first responses and PPR values were measured for each stimulation in each pharmacological condition and compared statistically (using a Mann–Whitney U test, as the normal distribution hypothesis is not tenable for ratios of variables). Average data from different neurons were compared using a Wilcoxon matched-pairs signed rank test.

Spontaneous GABAergic IPSPs were defined as fast upward deflections exceeding a threshold of twice the standard deviations (SD) of the baseline noise. At least five consecutive minutes were analyzed for each pharmacological condition. In each experiment, the average event amplitude and average inter-event interval observed in control and in the presence of drug(s) were compared statistically. The analysis of spontaneous IPSPs was performed using Spike 2 Software (C.E.D.).

### Drugs

All drugs were obtained from Tocris Biosciences (UK) and were bath-applied by at the concentrations mentioned in the “Results” Section.

### Data Analysis

Data were acquired using Signal (2.9) Software and a Micro 1401 data acquisition unit (C.E.D.). Off-line data analysis was carried out with Signal and Spike 2 software.

All results are expressed as mean ± SD. Student’s unpaired *t*-test was used for statistical comparisons, except for PPRs (see below). Statistical significance was accepted if *p* < 0.05.

## Results

### Cholinergic Control of LTS Interneurons

LTS interneurons are relatively rare in the striatum and cannot be visually identified in wild-type animals, making systematic electrophysiological investigations of these cells problematic in wild type animals (Beatty et al., 2012). Therefore, we used NPY-GFP mice in which LTS interneurons express GFP and can be subsequently identified based on their typical electrophysiological properties (See “Materials and Methods” Section; Figure 1B). A total of 75 NPY-GFP mice (both sexes) aged 25 ± 5 days were used in this study. Whole-cell recordings were obtained from 86 GFP-positive striatal LTS neurons. Of these, 82 were spontaneously active in the absence of any injected current, with an average firing frequency of 4.4 ± 1.7 Hz.

### Effects of Cholinergic Receptor Antagonists on LTS Interneurons

Cholinergic interneurons are the main source of ACh in the striatum (Dautan et al., 2014). As most cholinergic interneurons are spontaneously active in brain slices (Wilson, 2005), they are likely to generate a substantial tonic level of ACh in the striatum. We therefore used antagonists of muscarinic and nicotinic receptors to study how endogenous ACh affects LTS interneurons.

**Figure 2:**
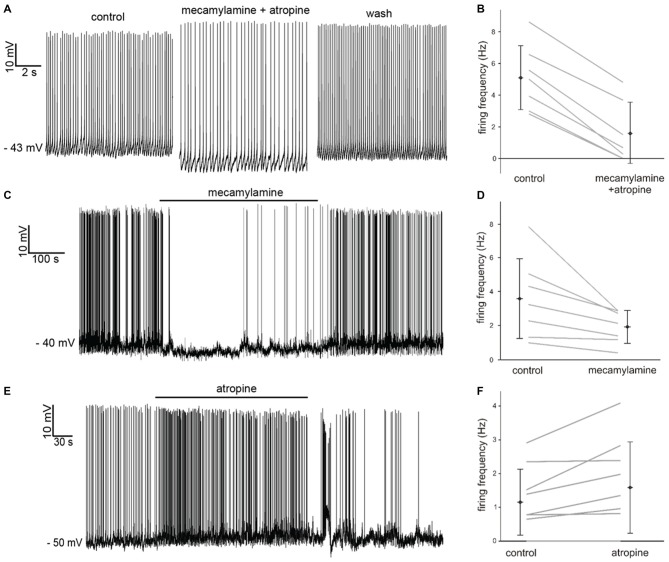
**Effects of nicotinic and muscarinic receptor antagonists on LTS interneurons. (A)** Co-application of mecamylamine and atropine produced a hyperpolarization accompanied by decreased firing in a LTS interneuron. These effects were reversed after washout. **(B)** Average spontaneous firing frequency observed in seven LTS interneurons before and after co-application of mecamylamine and atropine. **(C)** Application of mecamylamine produced a reversible hyperpolarization accompanied by cessation of spontaneous firing in a LTS interneuron. **(D)** Average spontaneous firing frequency observed in seven LTS interneurons before and after co-application of mecamylamine. **(E)** Application of atropine produced a depolarization and an increase in spontaneous firing frequency in a LTS interneuron. These effects were reversed after washout. **(F)** Average spontaneous firing frequency observed in nine LTS interneurons before and after co-application of atropine. Two LTS interneurons did not fire in either condition.

Simultaneous application of the broad spectrum nicotinic receptor antagonist mecamylamine hydrochloride (10 μM) and the broad spectrum muscarinic receptor antagonist atropine (20 μM) had an inhibitory influence, causing significant (*p* < 0.05) increases in the average inter-spike-interval (ISI) in 7 out of 7 LTS interneurons recorded in whole-cell configuration (Figure 2A). The effects of mecamylamine and atropine on spontaneous firing frequency in individual experiments are plotted in Figure 2B.

In order to dissect out the contribution of each receptor class to the observed effects, we then applied the two antagonists individually. Mecamylamine also produced a significant (*p* < 0.001) increase in average ISI in 7 out of 7 LTS interneurons, as shown in Figure 2C. The effects of mecamylamine on spontaneous firing frequency in individual experiments are plotted in Figure 2D.

Bath-application of atropine produced opposite effects on LTS interneurons. Atropine caused significant (*p* < 0.05) decreases in the average ISI in 5/9 LTS interneurons (Figure 2E), while the ISI was not significantly affected in the remaining 4/9 cells. The effects of atropine on spontaneous firing frequency in individual experiments are plotted in Figure 2F.

We concluded from these pharmacological experiments that a strong cholinergic tone is present in the striatum and has a net excitatory influence on LTS interneurons.

### Influence of Tonic GABA on LTS Interneurons

**Figure 3:**
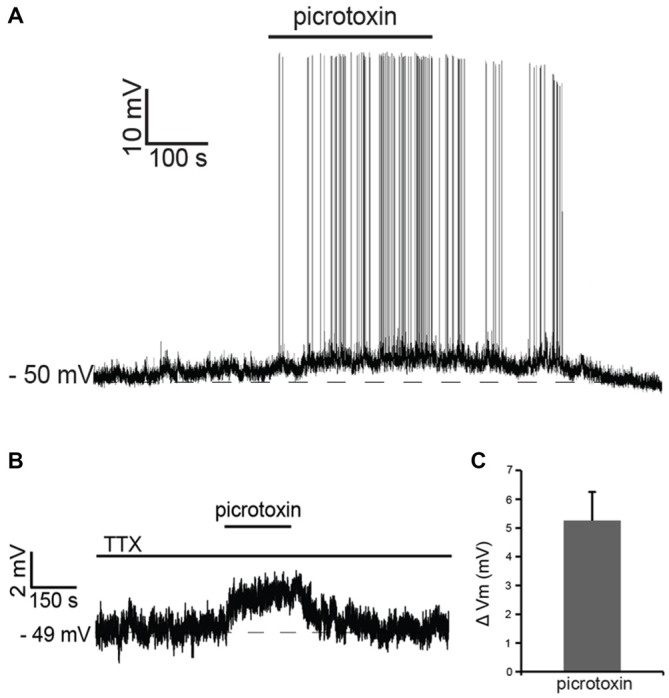
**Tonic GABA inhibits LTS interneurons. (A)** In a LTS interneuron that was not spontaneously active, application of picrotoxin triggered a reversible depolarization accompanied by action potential firing. **(B)** In another LTS interneuron, in the presence of tetrodotoxin (TTX), application of picrotoxin caused a reversible depolarization. **(C)** Average changes in membrane potential observed in 15 LTS interneurons after application of picrotoxin in the presence of TTX.

Recent studies have shown that tonic GABA present in the striatum affects the membrane potential of striatal projection neurons and that the activation of muscarinic and nicotinic receptors modulates this tonic GABAergic influence (Ade et al., 2008; Luo et al., 2013). In order to assess the possible contribution of GABA to the cholinergic effects observed in LTS interneurons, we initially studied the influence of tonic GABA on these cells. Bath application of the GABA_A_ antagonist picrotoxin (100 μM) had a strong excitatory influence, causing an increase in firing frequency in 6 out of 6 LTS interneurons tested. Of these cells, four were spontaneously active in control solution; in these cells, the average ISI increased significantly (*p* < 0.05) in the presence of picrotoxin. The remaining two neurons became spontaneously active in the presence of picrotoxin (Figure 3A).

In the presence of the sodium channel blocker tetrodotoxin citrate (TTX; 1 μM), picrotoxin evoked reversible depolarizations in 14 out of 15 LTS interneurons tested, as shown in the example of Figure 3B and in Figure 3C. We concluded that tonic GABA, released through both spike-dependent and spike-independent mechanisms, provides a strong inhibitory influence on LTS interneurons.

### Direct Effects of Cholinergic Antagonists on LTS Interneurons

**Figure 4:**
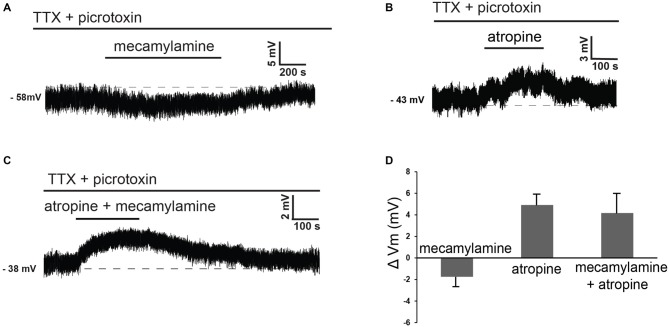
**Nicotinic and muscarinic antagonists affect LTS interneurons in the presence of TTX and picrotoxin. (A)** In the presence of TTX and picrotoxin, mecamylamine reversibly hyperpolarized a LTS interneuron. **(B)** In the presence of TTX and picrotoxin, atropine reversibly depolarized a LTS interneuron (different cell from panel **A**). **(C)** In the presence of TTX and picrotoxin, co-application of mecamylamine and atropine caused a reversible depolarization in a LTS interneuron (different cell from panels **A,B**). **(D)** Average effects of mecamylamine alone (*n* = 4), atropine alone (*n* = 4) or co-application of mecamylamine and atropine (*n* = 4) on the membrane potential of LTS interneurons. Twelve different interneurons were used for these applications.

These observations suggested that at least part of the effects of cholinergic receptor antagonists on these cells could be due to modulation of tonic GABA levels. In order to dissect out the direct influence of endogenous ACh on LTS interneurons, we investigated the effects of nicotinic and/or muscarinic receptor blockers in the presence of both picrotoxin and TTX. Under these conditions, mecamylamine elicited hyperpolarizations in 5 out of 6 LTS interneurons (no effects were observed in the remaining one). Conversely, atropine caused depolarizations in 5/5 LTS interneurons. Examples of these effects are illustrated in Figures 4A,B. In contrast to the experiments carried out in control solution, in the presence of TTX and picrotoxin co-application of mecamylamine and atropine caused depolarizations in 3/5 LTS interneurons (no effects in the remaining two), as shown in the example of Figure 4C. The average effects of individual blockers and their co-application in the presence of picrotoxin and TTX are shown in Figure 4D.

The observation that co-application of nicotinic and muscarinic receptor antagonists had opposite effects in control solution and in the presence of TTX and picrotoxin, suggested that indirect cholinergic effects on GABA release played a major role in the cholinergic control of LTS interneurons.

### Cholinergic Control of GABAergic Events

While tonic GABA levels could not be directly measured, we investigated the effects of cholinergic antagonists on spontaneous and evoked GABAergic events detectable in LTS interneurons. In these experiments, membrane potentials were kept at hyperpolarized levels (between −79 and −90 mV) by steady current injections. Changes in membrane potential induced by cholinergic antagonists were compensated manually, so that the membrane potential was maintained at control level throughout the experiment.

**Figure 5:**
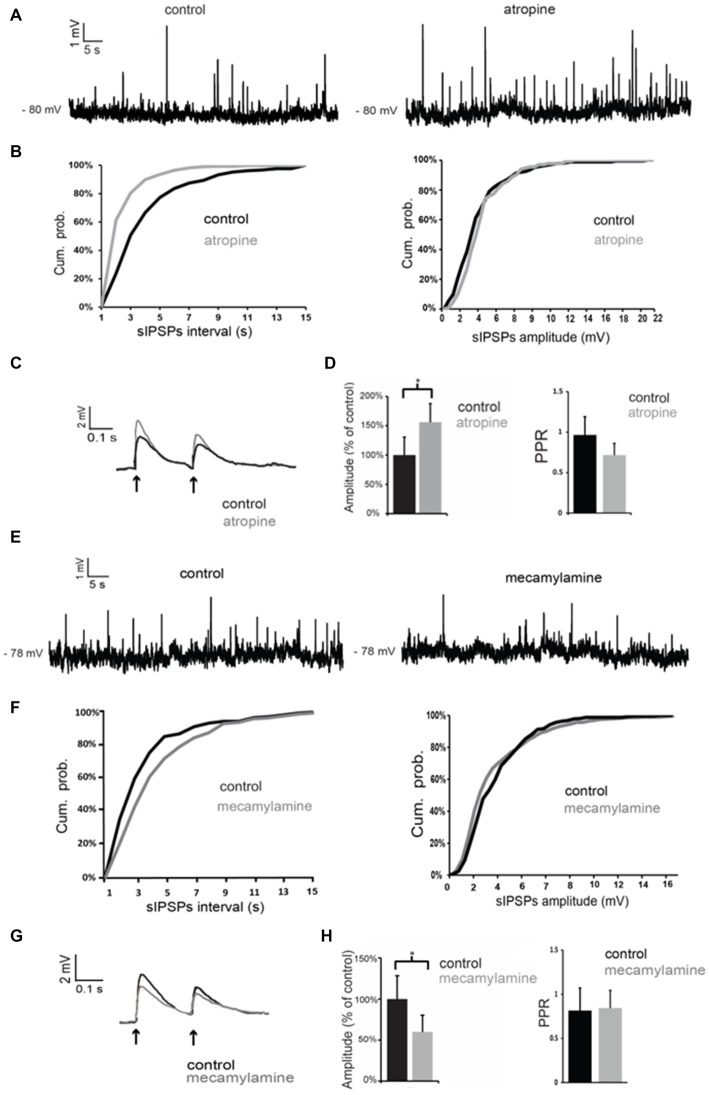
**Nicotinic and muscarinic antagonists affect GABAergic inputs to LTS interneurons. (A)** Atropine increased the frequency of spontaneous GABAergic events in a LTS interneuron. **(B)** Quantification of the effects of atropine on frequency and amplitude of spontaneous GABAergic events in the interneuron of **(A)**. The frequency, but not the amplitude, of spontaneous inhibitory postsynaptic potentials (IPSPs) was significantly (*p* < 0.05 for inter-event intervals) increased by atropine. **(C)** The amplitude of evoked GABAergic PSPs was significantly increased by atropine in a LTS interneuron. Traces are average of five consecutive events evoked by paired-pulse stimulation (200 ms intervals) delivered every 10 s. **(D)** Average effects of atropine on the amplitude of the first evoked GABAergic PSP and on Paired-pulse ratios (PPR). For each experiment, the amplitude of GABA responses in the presence of atropine was expressed as percentage of average GABA response amplitude in control solution. Asterisk indicates statistical significance at *p* < 0.05. **(E)** Mecamylamine decreased the frequency of spontaneous GABAergic events in a LTS interneuron. **(F)** Quantification of the effects of mecamylamine on frequency and amplitude of spontaneous GABAergic events in the interneuron of **(A)**. The frequency, but not the amplitude, of spontaneous IPSPs was significantly (*p* < 0.05 for inter-event intervals) decreased by mecamylamine. **(G)** The amplitude of evoked GABAergic PSPs was significantly decreased by mecamylamine in a LTS interneuron. Traces are average of five consecutive events evoked by paired-pulse stimulation (200 ms intervals) delivered every 10 s. **(H)** Average effects of mecamylamine on the amplitude of the first evoked GABAergic PSP and on PPR. For each experiment, the amplitude of GABA responses in the presence of mecamylamine was expressed as percentage of average GABA response amplitude in control solution. Asterisk indicates statistical significance at *p* < 0.05.

Atropine significantly (*p* < 0.05 for inter-event intervals) increased the frequency of spontaneous GABAergic events without affecting their average amplitude in 5 out of 5 LTS interneurons (Figures 5A,B). On average, across all neurons tested, the inter-event interval decreased by 49 ± 7% in the presence of atropine.

GABAergic events evoked by paired-pulse stimulation were also significantly (*p* < 0.05) increased in amplitude in the presence of atropine in 4 out of 5 cells, as shown in the example of Figure 5C and in the plot of Figure 5D. The PPR was significantly (*p* < 0.05) reduced in the presence of atropine in 4/5 cells. However, average PPR values pooled from different neurons were not significantly different in control and in the presence of atropine (Figure 5D).

Conversely, mecamylamine significantly (*p* < 0.05) reduced the frequency of the spontaneous GABAergic events without affecting their average amplitude in 4/4 LTS interneurons (Figures 5E,F). On average, across all neurons tested, the inter-event interval increased by 111 ± 4% in the presence of mecamylamine.

In the presence of mecamylamine (Figures 5G,H) GABAergic evoked responses were also significantly (*p* < 0.05) reduced in amplitude in 4/4 neurons (Figures 5G,H). PPR was not significantly affected by mecamylamine in any of these cells (average values are shown in Figure 5H).

We concluded that tonic activation of nicotinic receptors by endogenous ACh has a facilitatory action on GABA inputs to LTS interneurons, while activation of muscarinic receptors has an inhibitory influence.

**Figure 6:**
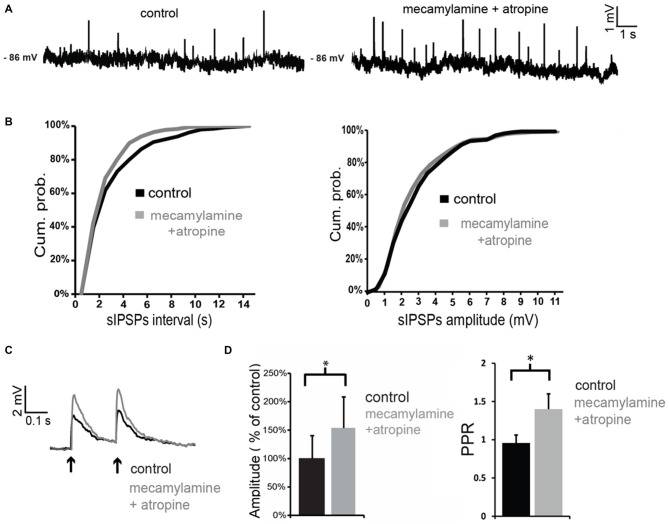
**Co-application of nicotinic and muscarinic antagonists increases GABAergic inputs to LTS interneurons. (A)** Co-application of mecamylamine and atropine increased the frequency of spontaneous GABAergic events in a LTS interneuron. **(B)** Quantification of the effects of co-application of mecamylamine and atropine on frequency and amplitude of spontaneous GABAergic events in the interneuron of panel **(A)**. The frequency, but not the amplitude, of spontaneous IPSPs was significantly (*p* < 0.05 for inter-event intervals) increased by the antagonists. **(C)** The amplitude of evoked GABAergic IPSPs was significantly increased by co-application of mecamylamine and atropine in a LTS interneuron. Traces are average of five consecutive events evoked by paired-pulse stimulation (200 ms intervals) delivered every 10 s. **(D)** Average effects of co-application of mecamylamine and atropine on the amplitude of the first evoked GABAergic PSP and on PPR. For each experiment, the amplitude of GABA responses in the presence of atropine and mecamylamine was expressed as percentage of average GABA response amplitude in control solution. Asterisks indicate statistical significance at *p* < 0.05.

When antagonists of muscarinic and nicotinic receptors were applied simultaneously, the net effect on spontaneous GABA events was a significant increase in frequency (but not in amplitude) in 3/4 LTS interneurons (as shown in the example of Figures 6A,B). On average, across all neurons tested, the inter-event interval decreased by 77 ± 8% in the combined presence of atropine and mecamylamine. Co-application of muscarinic and nicotinic receptor antagonists also significantly (*p* < 0.05) increased the amplitude of evoked GABA responses in 4/4 LTS interneurons (Figures 6C,D). This was accompanied in all neurons by a significant (*p* < 0.05) increase in the PPR (Figure 6C). Average effects of atropine and mecamylamine on PPR for all neurons tested are shown in Figure 6D.

The observation that co-application of mecamylamine and atropine increases GABA events suggests that tonic ACh has a net inhibitory effect on GABA release onto LTS interneurons, even though the origin of the GABAergic inputs to LTS interneurons is complex and could not be dissected out. Furthermore, these results suggest that the hyperpolarizing effects caused by co-application of mecamylamine and atropine in control solution (but not in TTX and picrotoxin) are due to an increase in tonic GABA.

### Nitrergic Control of Cholinergic Interneurons

**Figure 7:**
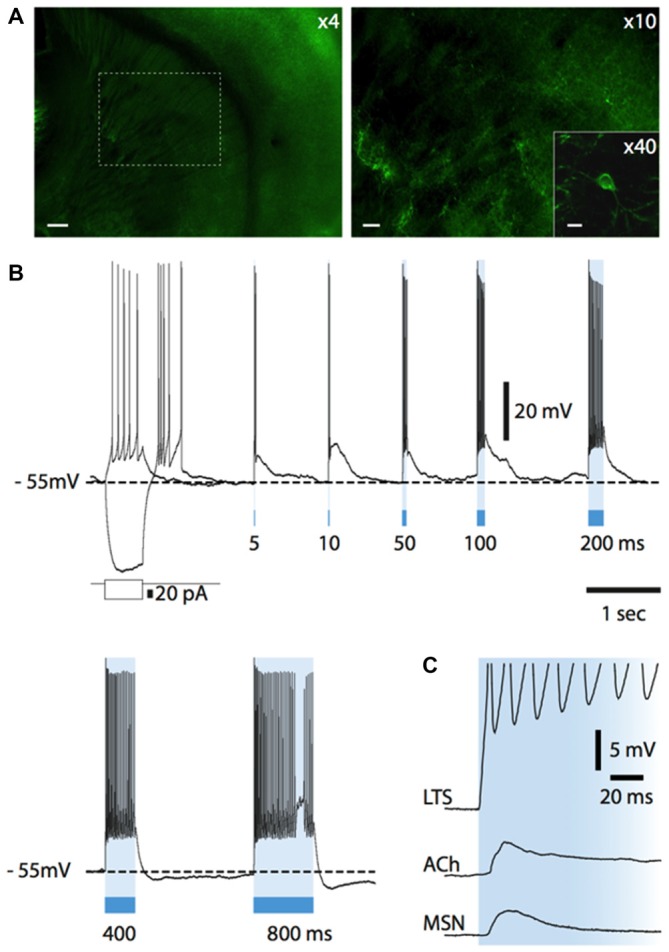
**Light-evoked responses of striatal neurons in Som-ChR2 mice. (A)** Fluorescent photomicrography of a Som-ChR2 mouse brain slice, comprising striatum, corpus callosum and cortex (left). In the right panel, the part of striatum contained in the dashed square in the left panel is shown at higher magnification. Several eYFP-positive somata and their extensive arborizations can be identified. The inset shows an eYFP-positive cell and its primary dendrites. Calibration bars: 200 μm (left panel), 60 μm (right panel) and 20 μm (inset). **(B)** Responses of an eYFP-positive neuron to a negative and a positive current step (superimposed) and to several light pulses of increasing length. A rebound low-threshold calcium spike, accompanied by several action potentials, was present after termination of the negative current pulse. Light pulses of increasing duration (5–800 ms) were applied after the negative current step. Even the shortest light pulse (5 ms) elicited a strong supra-threshold response in the interneuron. LTSs developing after termination of the light stimulus are evoked by 5, 10, 50 and 100 ms pulses. Longer light pulse lengths evoke prolonged action potential firing. **(C)** In an eYFP-positive LTS interneuron, a light pulse evoked a short-latency (<1 ms), persistent supra-threshold depolarization. Other neuronal types (cholinergic interneurons, MSNs) tested with the same protocol displayed much smaller and transient initial responses with a substantially longer latency (>5 ms). Resting potentials were −78 mV (MSN), −64 mV (cholinergic interneuron), −64 mV (LTS interneuron).

Having elucidated how endogenous ACh affects the activity of LTS interneurons, we investigated if LTS interneurons could in turn control cholinergic interneurons. For this study, we used a strain of transgenic mice in which somatostatin-expressing neurons selectively express channelrhodopsin and eYFP (see "Materials and Methods" Section for details). A total of 29 ChR2-SOM mice (both males and females) aged 28 ± 6 days were used in this study. In these mice, expression of eYFP in the striatum was limited to a minority of neurons, in which fluorescence was detected in cell bodies and proximal dendrites, as shown in Figure 7A. A fluorescence background of variable intensity was also present in the cortex and in the striatum, but not in the corpus callosum, and was presumably due to eYFP-positive fibers that were too small to be individually identified (Figure 7A). Whole-cell recordings were carried out from 40 cholinergic interneurons. Of these, 38 were spontaneously active, with an average firing frequency of 2.3 ± 1.2 Hz.

### Influence of Tonic Somatostatin, NPY and Nitric Oxide on Cholinergic Interneurons

LTS interneurons express GABA, somatostatin and NPY, in addition to nitric oxide synthase (Kawaguchi, 1993; Tepper et al., 2010). As most LTS interneurons are spontaneously active and therefore likely to generate tonic levels of these neurotransmitters, we initially studied the effects of antagonists selective for their receptors on the activity of cholinergic interneurons. However, as the effects of blocking GABA receptors on cholinergic interneurons have already been extensively described (Bennett and Wilson, 1998; Bonsi et al., 2003), we did not carry out experiments with GABA receptor antagonists.

Bath application of the broad spectrum NPY receptor antagonist PD 160170 (20 μM) failed to affect the spontaneous firing or membrane potential of cholinergic interneurons (*n* = 4; average ISI was 0.8 ± 0.5 s in control and 0.7 ± 0.5 s in the presence of PD190170). Similarly, no effects were observed on cholinergic interneurons when the broad-spectrum somatostatin receptor antagonist cyclosomatostatin (1 μM) was applied (*n* = 4; average ISI in control was 1.5 ± 0.6 s in control and 1.4 ± 0.5 s in the presence of cyclosomatostatin). Application of the nitric oxide synthase inhibitor L-NAME (100 μM) also failed to affect the membrane properties of cholinergic interneurons (*n* = 4; average ISI was 1.1 ± 0.2 s in control and 1.0 ± 0.1 s in the presence of L-NAME). We concluded that tonic levels of NPY, somatostatin and nitric oxide have little influence on the excitability of cholinergic interneurons.

### Influence of Phasic Activation of LTS Interneurons on Cholinergic Interneurons

**Figure 8:**
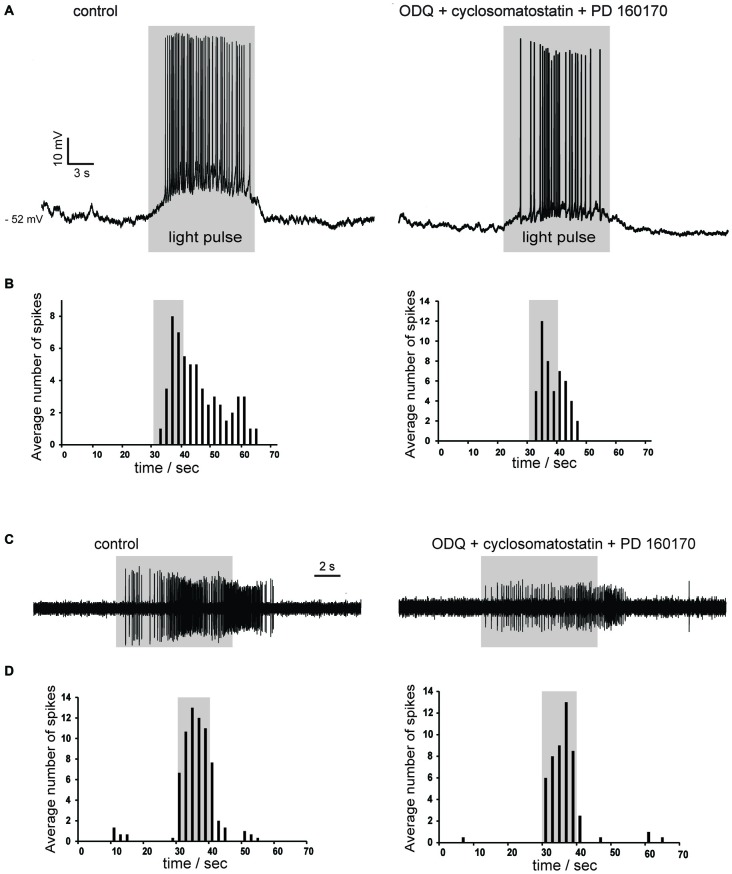
**Photostimulation evokes slow depolarizations in cholinergic interneurons in Som-ChR2 mice. (A)** In a whole-cell experiment, a 10 s light pulse evoked a slowly-developing depolarization accompanied by robust firing in a cholinergic interneuron that was not spontaneously active (left). No early fast responses to light pulses were observed in this particular neuron. The slow depolarizations evoked by photostimulation persisted in the simultaneous presence of ODQ, cyclosomatostatin and PD 160170 (right). **(B)** Distribution of action potentials in the cholinergic interneuron of **(A)** during photostimulation in control solution (left) or in the presence of ODQ, cyclosomatostatin and PD 160170 (right). Spikes were detected in the 30 s preceding the light pulse, during the 10 s light pulse, and in the 30 s following the light pulse over five consecutive presentations of the photostimulation. The average number of spikes were calculated for adjacent 2 s time bins. Firing probability remained elevated for several seconds after the end of the light pulse. **(C)** A different cholinergic interneuron was recorded in cell-attached configuration. The neuron was sporadically active, but light stimulation (10 s) evoked a strong burst of spikes that persisted for several seconds after the pulse in control solution (left). A similar pattern of action potentials was elicited by light pulses in the presence of ODQ, cyclosomatostatin and PD 160170 (right). The decrease in spike amplitude is likely due to slight changes in the electrode-membrane seal during the cell-attached experiment. **(D)** Temporal distribution of spikes before, during and after light pulses for the experiment of **(C)**. Data were averaged over five consecutive presentations of photostimulations, in control solution (left) and in the presence of ODQ, cyclosomatostatin and PD 160170 (right).

**Figure 9:**
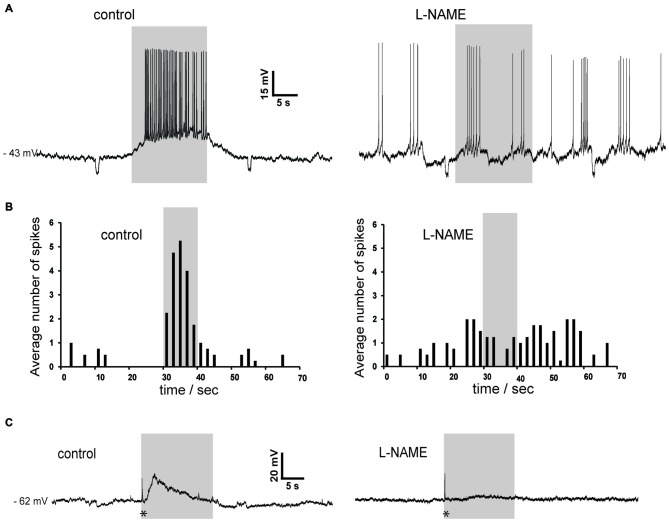
**Slow depolarizations of cholinergic interneurons induced by photostimulation are blocked by the NOS inhibitor L-NAME in Som-ChR2 mice. (A)** In a sporadically active cholinergic interneuron recorded in whole-cell configuration, light stimulation caused a robust depolarization accompanied by action potentials (left). Subsequently, application of L-NAME completely abolished the ability of light pulses to affect the timing of action potentials. Note that the spontaneous firing pattern has changed during the experiment, a phenomenon often observed in cholinergic interneurons. Small negative deflections were caused by current steps (10 pA, 0.5 s) injected every 30 s. **(B)** Distribution of action potentials in the experiment of panel **(A)** in control solution (left) and in the presence of L-NAME (right). Spike numbers were averaged over five consecutive presentations of the photostimulation. **(C)** In another neuron that was not spontaneously active, photostimulation caused an initial fast response (asterisk), followed by a slowly-developing subthreshold depolarization in control solution (left). In the presence of L-NAME, the early fast response (asterisk) was unchanged while the slow depolarization was abolished (right).

Nitric oxide release is thought to require large and prolonged depolarizations of nitric oxide synthase-expressing neurons (Garthwaite, 2008). Therefore, we tested the effects of photostimulation of the striatum in Som-ChR2 mice, which is expected to elicit selective depolarization of LTS interneurons (Rafalovich et al., 2015). Consistent with this notion, eYFP-positive neurons displayed membrane properties typical of LTS interneurons (Figure 7B). Furthermore, in these cells, blue light pulses (5 ms–10 s) elicited short latency (<1 ms), very large (>20 mV) depolarizations leading to intense action potential firing, as shown in Figures 7B,C. This was in sharp contrast with the responses to blue light observed in MSNs, in which much smaller (<5 mV), subthreshold responses were observed in 11/12 cases (Figure 7C). The latency of these responses in MSNs was always >5 ms and they were strongly reduced by application of the GABA_A_ receptor antagonist picrotoxin. We therefore attributed these responses to synaptic GABA released from LTS interneurons excited by blue light. In some cholinergic interneurons hyperpolarized by current injections (8/12), an early response to blue light pulses, similar to that observed in MSNs, was also observed, as shown in Figures 7C, 9C. Furthermore, in cholinergic interneurons (but never in MSNs), prolonged (10 s) light pulses also elicited slower depolarizing effects that were often accompanied by strong action potential firing, as shown in the examples of Figures 8, 9. Such slow depolarizing responses were elicited by 10 s blue light pulses in 15 out of 24 cholinergic interneurons (63%) in whole-cell experiments. They had peak amplitudes >10 mV and often outlasted the light pulses, as did the associated action potentials (Figures 8, 9). In agreement with the effects observed in whole-cell recordings, photostimulation produced delayed bursts of action potentials in 3 out of 3 cell-attached recordings from cholinergic interneurons (Figures 8C,D). In the example of Figures 8C,D, the action potentials continued for several seconds after the end of a light pulse. Shorter light pulses (50–1000 ms) failed to elicit measurable responses in 7/7 cholinergic interneurons, suggesting that prolonged depolarization of LTS interneurons was required for these effects.

### Pharmacology of Slow Depolarizations in Cholinergic Interneuron Response

In order to establish their pharmacology, we tested if these slow depolarizations were blocked by antagonists of the receptors likely to be activated by LTS interneurons. The slow depolarizations were not affected by application of cyclosomatostatin (*n* = 4) or PD 160170 (*n* = 4), showing that somatostatin and NPY were not responsible for these responses. Nitric oxide acts in the striatum through both guanylate cyclase (GC)-dependent and GC-independent mechanisms (Hartung et al., 2011). We probed the involvement of GC by applying the selective GC inhibitor ODQ (10 μM; *n* = 7). However, ODQ did not affect the light-induced slow depolarizations (*n* = 7). These results are illustrated by the experiments of Figures 8A,C, in which the excitatory responses to blue light persisted in the simultaneous presence of cyclosomatostatin, PD 160170 and ODQ.

To further investigate the possible involvement of nitric oxide, we carried out experiments in which L-NAME was applied. As shown in the two representative examples of Figure 9, L-NAME completely abolished the slow depolarizing responses induced by blue light in cholinergic interneurons (*n* = 4), regardless of whether these responses were accompanied by action potentials or not. During light pulses, average ISI was 0.30 ± 0.04 s in control solution and 1.7 ± 0.6 s in the presence of L-NAME (*p* < 0.001). We concluded that synchronized depolarization of LTS interneurons in the striatum causes a strong excitation of cholinergic interneurons, which is mediated by nitric oxide but does not require GC activation.

## Discussion

This study investigated the interactions between LTS and cholinergic interneurons, the two interneuronal types that are spontaneously active in the striatum. Our data indicate that strong mutual excitation exists between these cell types.

**Figure 10:**
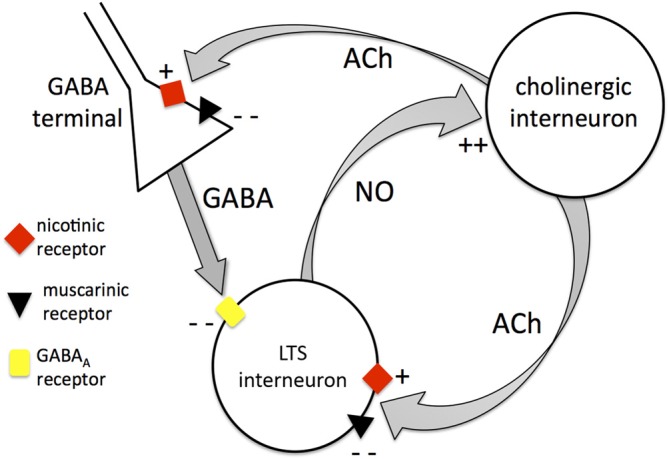
**Mutual control between cholinergic interneurons and LTS interneurons.** Diagram summarizing the interactions between cholinergic interneurons and LTS interneurons. The cholinergic interneurons modulate LTS interneurons through Acetylcholine (ACh) both directly and indirectly. Directly, ACh can activate LTS interneurons through nicotinic receptors or inhibit LTS interneurons through muscarinic receptors (the latter being the dominant effect). Indirectly, ACh can increase GABA release from unidentified GABA terminals through nicotinic action leading to inhibition of LTS interneurons. On the other hand, ACh can inhibit GABA release through muscarinic action (this is the dominant effect). The net overall influence of cholinergic interneurons on LTS interneurons is excitatory. LTS interneurons in turn release nitric oxide that depolarizes cholinergic interneurons, providing the basis for a mutually excitatory positive feedback mechanism between the two interneuronal types.

The significant excitatory influence exerted by tonic endogenous ACh on LTS interneurons is clearly demonstrated by the strong hyperpolarizations caused by pharmacological block of nicotinic and muscarinic receptors. Our experiments showed that this results from a combination of effects, involving both receptor classes. Each of these receptor types affects the LTS interneurons both directly (by modulating their intrinsic excitability) and indirectly (by modulating tonic GABA levels). Nicotinic receptors have a direct excitatory influence on LTS interneurons, but they also increase the inhibitory GABA inputs impinging on these cells. Muscarinic receptors inhibit the LTS interneurons directly, but at the same time reduce their GABAergic inputs. The net effect of tonic ACh on GABAergic activity is inhibitory, as co-application of nicotinic and muscarinic antagonists increases the frequency of detectable GABAergic events. If we extrapolate these results to tonic GABA, then the cholinergic reduction of GABA levels is expected to exert a net excitatory influence on LTS interneurons (through inhibition of their synaptic inhibition). On the other hand, in terms of direct effects, the net action of tonic activation of muscarinic and nicotinic is inhibitory, as in the presence of TTX and picrotoxin co-application of the two antagonists depolarizes the neurons. Under these conditions, ambient ACh presumably resulted from spike-independent exocytosis of ACh vesicles from cholinergic interneuron terminals. On balance, the indirect excitatory effects of ACh prevailed on the direct inhibitory ones. These effects are summarized in Figure 10. It should be noted that the identity of the GABA terminals affecting LTS interneurons has not been unraveled in this study, and a number of possible sources exist in the striatum (Szydlowski et al., 2013).

This prevalence is likely to be even more dramatic *in vivo*. Most striatal GABAergic neurons are not spontaneously active in brain slices (Tepper et al., 2010). Therefore, the GABA dependent excitatory influence of ACh on LTS interneurons can be expected to be even larger in the intact brain, where GABA levels are expected to be larger as striatal GABAergic cells are activated by cortical and thalamic inputs (Klaus et al., 2011). The ability of cholinergic interneurons to increase the excitability of LTS interneurons is likely to be important for the dynamics of the local neuronal networks, not only in terms of the mutual excitatory interactions demonstrated in this study, but also because LTS interneurons mediate feed-forward inhibition of striatal projection neurons (Koós and Tepper, 1999) and are also likely to release somatostatin (Kawaguchi, 1993), that is involved in the modulation of inhibitory interaction of projection neurons (López-Huerta et al., 2012). Furthermore, cholinergic modulation of LTS interneurons is potentially relevant for the control of neurovascular coupling, which is believed to be mediated, at least in part, by neuronally produced nitric oxide (Laranjinha et al., 2012).

The changes in spontaneous GABAergic event frequency, but not their amplitude, suggests that both muscarinic and nicotinic receptors acted presynaptically to modulate GABA release, as shown in previous studies (Luo et al., 2013; Yamamoto et al., 2013), although the contribution of postsynaptic mechanisms (including changes in chloride intracellular concentration) cannot be ruled out. While PPRs of evoked GABAergic responses were affected, the interpretation of this observation is not straightforward, as the electrical stimuli delivered locally is expected to activate cholinergic axons in addition to GABAergic ones, giving rise to a situation far more complex than that of traditional experiments with exogenous agonists.

We initially used a similar pharmacological approach, based on the application of selective receptor antagonists, to investigate how the LTS interneurons (which are also spontaneously active in brain slices) affected cholinergic interneurons. However, blocking somatostatin and NPY receptors or preventing nitric oxide production did not elicit detectable effects in cholinergic interneurons. This suggests that either the spontaneous action potentials of the LTS interneurons are unable to release these neurotransmitters in concentrations large enough to influence the cholinergic interneurons, or that these neurotransmitters are not involved in the communication between these interneuronal classes. Neuronal nitric oxide synthases require significant increases in intracellular calcium to become activated, possibly accompanied by activation of NMDA receptors (Garthwaite, 2008). Thus, the relatively sparse action potentials generated by LTS interneurons in brain slices may not be sufficient for significant nitric oxide production. In order to be able to photo-stimulate LTS interneurons selectively, we crossed transgenic mice expressing Cre-dependent channelrhodopsin constructs with mice expressing Cre recombinase exclusively in neurons expressing somatostatin. A similar transgenic strategy was recently used to stimulate striatal LTS interneurons by Rafalovich et al. (2015). As expected, in the striatum of the resulting offspring, photostimulation produced fast and large depolarizations in LTS interneurons but not in MSNs or cholinergic interneurons. However, prolonged light pulses evoked slowly developing, sustained depolarizations of cholinergic interneurons, often accompanied by robust action potential firing. These excitatory responses can be safely attributed to nitric oxide, as they were entirely blocked by L-NAME, a selective nitric oxide synthase inhibitor, while they were not affected by somatostatin or NPY receptor antagonists. GABA, the remaining transmitter released by LTS interneurons, can also be ruled out, as it would produce hyperpolarizing responses in cholinergic interneurons under our recording conditions.

Interestingly, these nitrergic effects were not blocked by a glucocerebrosidase (GC) inhibitor. GC is the main known nitric oxide receptor and mediates nitrergic responses in several neuronal types (Garthwaite, 2008). Nevertheless, in another study, nitric oxide-mediated modulation of dopamine release in the striatum was also found to be independent of GC activation (Hartung et al., 2011), suggesting that alternative nitric oxide-receptors and associated biochemical pathways are present in this area. Further experiments will be required to identify the molecular cascades involved in the nitrergic excitation of cholinergic interneurons. A previous study, carried out in rats, found that nitric oxide donors depolarized cholinergic interneurons through GC activation (Centonze et al., 2001). It is likely that such exogenous applications produce a spatiotemporal profile of nitric oxide elevation that differs substantially from that caused by neuronal activation and therefore engage and/or desensitize different molecular targets.

It is remarkable that, in the present study, prolonged light-induced depolarizations of LTS interneurons were sufficient to release nitric oxide. This was not easy to predict, as nitric oxide synthase is often physically associated with NMDA receptors and is thought to require their activation to produce nitric oxide (Garthwaite, 2008). However, NMDA receptor activation is unlikely to have occurred substantially in our experimental conditions. Further experiments will be required to reveal if, and under which conditions, nitric oxide synthase can operate in LTS interneurons independently of NMDA receptors. The observation that light pulses up to 1 s in duration did not elicit measurable effects in cholinergic interneurons suggests that nitric oxide is unlikely to be released as a result of phasic excitatory signals to LTS interneurons and may rather take place in response to slower signals provided by neuromodulators such as dopamine, that has a strong excitatory influence on these neurons (Centonze et al., 2002). It is also possible that in the presence of NMDA receptor activation, nitric oxide release can take place on a faster timescale.

The present experiments revealed for the first time the presence, time course and extent of the excitation of cholinergic interneurons caused by endogenous, neuronally released nitric oxide. Nitrergic depolarizations usually peaked in the first few seconds of a photostimulation pulse and were able to evoke intense firing even in sporadically active or quiescent cholinergic interneurons. In some cases the depolarizations (and occasionally the firing) outlasted the light pulses. Clearly, these relatively slow responses are unlikely to partake in very fast neuronal processing and are rather expected to affect the local network dynamics over prolonged periods. Indeed, the complex communication between LTS and cholinergic interneurons (summarized in Figure 10 and including both direct and indirect mechanism) is likely to provide the cellular basis for a positive-feedback, mutually excitatory interaction, in which the two neuronal types may support each other in a protracted state of increased excitation. The functional significance of this configuration will have to be established with *in vivo* experiments. It is tempting to speculate that a state of heightened excitability of the two tonically active interneurons may provide an enhanced background for the firing pause that follows rewarding stimuli (Joshua et al., 2008), thus increasing the contrast of the response to such stimuli.

Given the ability of thalamic fibers to activate cholinergic interneurons (Ding et al., 2010), it is possible that an “up-state” of cholinergic and LTS interneurons is initiated by co-ordinated thalamic inputs and then self-maintained until a sufficiently large phasic inhibitory input is conveyed to either group of cells (or both). We have shown that serotonin excites cholinergic interneurons but it inhibits LTS interneurons (Blomeley and Bracci, 2005; Cains et al., 2012). It is tempting to speculate that serotonin, which is released in the striatum mainly by afferents from the raphe nucleus, may provide a mechanism to de-couple the two interneuronal classes, preventing their mutual excitation and biasing the local network towards cholinergic modulation.

## Author Contributions

RE and NV designed and carried out experiments, analyzed data and contributed to writing the manuscript. EB designed experiments, analyzed data and contributed to writing the manuscript.

## Conflict of Interest Statement

The authors declare that the research was conducted in the absence of any commercial or financial relationships that could be construed as a potential conflict of interest.

## References

[B1] Ade K. K. Janssen M. J. Ortinski P. I. Vicini S. (2008). Differential tonic GABA conductances in striatal medium spiny neurons. J. Neurosci. 28, 1185–1197. 10.1523/jneurosci.3908-07.2008 18234896 PMC6671393

[B2] Apicella P. (2007). Leading tonically active neurons of the striatum from reward detection to context recognition. Trends Neurosci 30, 299–306. 10.1016/j.tins.2007.03.011 17420057

[B3] Balleine B. W. Delgado M. R. Hikosaka O. (2007). The role of the dorsal striatum in reward and decision-making. J. Neurosci. 27, 8161–8165. 10.1523/jneurosci.1554-07.2007 17670959 PMC6673072

[B4] Beatty J. A. Sullivan M. A. Morikawa H. Wilson C. J. (2012). Complex autonomous firing patterns of striatal low-threshold spike interneurons. J. Neurophysiol. 108, 771–781. 10.1152/jn.00283.2012 22572945 PMC3424086

[B5] Bennett B. D. Callaway J. C. Wilson C. J. (2000). Intrinsic membrane properties underlying spontaneous tonic firing in neostriatal cholinergic interneurons. J. Neurosci. 20, 8493–8503. 11069957 10.1523/JNEUROSCI.20-22-08493.2000 PMC6773196

[B301] Bennett B. D. Wilson C. J. (1998). Synaptic regulation of action potential timing in neostriatal cholinergic interneurons. J. Neurosci. 18, 8539–8549. 9763496 10.1523/JNEUROSCI.18-20-08539.1998 PMC6792851

[B6] Blomeley C. Bracci E. (2005). Excitatory effects of serotonin on rat striatal cholinergic interneurones. J. Physiol. 569, 715–721. 10.1113/jphysiol.2005.098269 16269435 PMC1464279

[B7] Blomeley C. P. Bracci E. (2011). Opioidergic interactions between striatal projection neurons. J. Neurosci. 31, 13346–13356. 10.1523/jneurosci.1775-11.2011 21940429 PMC3781771

[B8] Blomeley C. P. Cains S. Smith R. Bracci E. (2011). Ethanol affects striatal interneurons directly and projection neurons through a reduction in cholinergic tone. Neuropsychopharmacology 36, 1033–1046. 10.1038/npp.2010.241 21289603 PMC3077272

[B9] Blomeley C. P. Kehoe L. A. Bracci E. (2009). Substance P mediates excitatory interactions between striatal projection neurons. J. Neurosci. 29, 4953–4963. 10.1523/jneurosci.6020-08.2009 19369564 PMC6665341

[B302] Bonsi P. Florio T. Capozzo A. Pisani A. Calabresi P. Siracusano A.. (2003). Behavioural learning-induced increase in spontaneous GABA_A_-dependent synaptic activity in rat striatal cholinergic interneurons. Eur. J. Neurosci. 17, 174–178. 10.1046/j.1460-9568.2003.02410.x 12534982

[B10] Bracci E. Centonze D. Bernardi G. Calabresi P. (2003). Voltage-dependent membrane potential oscillations of rat striatal fast-spiking interneurons. J. Physiol. 549, 121–130. 10.1113/jphysiol.2003.040857 12665602 PMC2342923

[B11] Cains S. Blomeley C. P. Bracci E. (2012). Serotonin inhibits low-threshold spike interneurons in the striatum. J. Physiol. 590, 2241–2252. 10.1113/jphysiol.2011.219469 22495583 PMC3424750

[B12] Centonze D. Bracci E. Pisani A. Gubellini P. Bernardi G. Calabresi P. (2002). Activation of dopamine D_1_-like receptors excites LTS interneurons of the striatum. Eur. J. Neurosci. 15, 2049–2052. 10.1046/j.1460-9568.2002.02052.x 12099911

[B13] Centonze D. Gubellini P. Bernardi G. Calabresi P. (1999). Permissive role of interneurons in corticostriatal synaptic plasticity. Brain Res. Brain Res. Rev. 31, 1–5. 10.1016/s0165-0173(99)00018-1 10611492

[B14] Centonze D. Pisani A. Bonsi P. Giacomini P. Bernardi G. Calabresi P. (2001). Stimulation of nitric oxide—cGMP pathway excites striatal cholinergic interneurons via protein kinase G activation. J. Neurosci. 21, 1393–1400. 11160411 10.1523/JNEUROSCI.21-04-01393.2001 PMC6762226

[B15] Dautan D. Huerta-Ocampo I. Witten I. B. Deisseroth K. Bolam J. P. Gerdjikov T.. (2014). A major external source of cholinergic innervation of the striatum and nucleus accumbens originates in the brainstem. J. Neurosci. 34, 4509–4518. 10.1523/jneurosci.5071-13.2014 24671996 PMC3965779

[B16] Ding J. B. Guzman J. N. Peterson J. D. Goldberg J. A. Surmeier D. J. (2010). Thalamic gating of corticostriatal signaling by cholinergic interneurons. Neuron 67, 294–307. 10.1016/j.neuron.2010.06.017 20670836 PMC4085694

[B17] Doig N. M. Magill P. J. Apicella P. Bolam J. P. Sharott A. (2014). Cortical and thalamic excitation mediate the multiphasic responses of striatal cholinergic interneurons to motivationally salient stimuli. J. Neurosci. 34, 3101–3117. 10.1523/jneurosci.4627-13.2014 24553950 PMC3931511

[B18] English D. F. Ibanez-Sandoval O. Stark E. Tecuapetla F. Buzsáki G. Deisseroth K.. (2011). GABAergic circuits mediate the reinforcement-related signals of striatal cholinergic interneurons. Nat. Neurosci. 15, 123–130. 10.1038/nn.2984 22158514 PMC3245803

[B19] Garthwaite J. (2008). Concepts of neural nitric oxide-mediated transmission. Eur. J. Neurosci. 27, 2783–2802. 10.1111/j.1460-9568.2008.06285.x 18588525 PMC2610389

[B20] Gerfen C. R. Surmeier D. J. (2011). Modulation of striatal projection systems by dopamine. Annu. Rev. Neurosci. 34, 441–466. 10.1146/annurev-neuro-061010-113641 21469956 PMC3487690

[B21] Hartung H. Threlfell S. Cragg S. J. (2011). Nitric oxide donors enhance the frequency dependence of dopamine release in nucleus accumbens. Neuropsychopharmacology 36, 1811–1822. 10.1038/npp.2011.62 21508928 PMC3154099

[B22] Ibáñez-Sandoval O. Tecuapetla F. Unal B. Shah F. Koos T. Tepper J. M. (2011). A novel functionally distinct subtype of striatal neuropeptide Y interneuron. J. Neurosci. 31, 16757–16769. 10.1523/jneurosci.2628-11.2011 22090502 PMC3236391

[B23] Joshua M. Adler A. Mitelman R. Vaadia E. Bergman H. (2008). Midbrain dopaminergic neurons and striatal cholinergic interneurons encode the difference between reward and aversive events at different epochs of probabilistic classical conditioning trials. J. Neurosci. 28, 11673–11684. 10.1523/jneurosci.3839-08.2008 18987203 PMC6671303

[B24] Kawaguchi Y. (1993). Physiological, morphological, and histochemical characterization of three classes of interneurons in rat neostriatum. J. Neurosci. 13, 4908–4923. 7693897 10.1523/JNEUROSCI.13-11-04908.1993 PMC6576359

[B25] Klaus A. Planert H. Hjorth J. J. Berke J. D. Silberberg G. Kotaleski J. H. (2011). Striatal fast-spiking interneurons: from firing patterns to postsynaptic impact. Front. Syst. Neurosci. 5:57. 10.3389/fnsys.2011.00057 21808608 PMC3139213

[B26] Koós T. Tepper J. M. (1999). Inhibitory control of neostriatal projection neurons by GABAergic interneurons. Nat. Neurosci. 2, 467–472. 10321252 10.1038/8138

[B27] Koos T. Tepper J. M. Wilson C. J. (2004). Comparison of IPSCs evoked by spiny and fast-spiking neurons in the neostriatum. J. Neurosci. 24, 7916–7922. 10.1523/jneurosci.2163-04.2004 15356204 PMC6729926

[B28] Laranjinha J. Santos R. M. Lourenço C. F. Ledo A. Barbosa R. M. (2012). Nitric oxide signaling in the brain: translation of dynamics into respiration control and neurovascular coupling. Ann. N. Y. Acad. Sci. 1259, 10–18. 10.1111/j.1749-6632.2012.06582.x 22758631

[B29] Logie C. Bagetta V. Bracci E. (2013). Presynaptic control of corticostriatal synapses by endogenous GABA. J. Neurosci. 33, 15425–15431. 10.1523/jneurosci.2304-13.2013 24068811 PMC3782622

[B30] López-Huerta V. G. Blanco-Hernández E. Bargas J. Galarraga E. (2012). Presynaptic modulation by somatostatin in the rat neostriatum is altered in a model of parkinsonism. J. Neurophysiol. 108, 1032–1043. 10.1152/jn.00244.2012 22623487

[B31] Luo R. Janssen M. J. Partridge J. G. Vicini S. (2013). Direct and GABA-mediated indirect effects of nicotinic ACh receptor agonists on striatal neurones. J. Physiol. 591, 203–217. 10.1113/jphysiol.2012.241786 23045343 PMC3630781

[B32] Pakhotin P. Bracci E. (2007). Cholinergic interneurons control the excitatory input to the striatum. J. Neurosci. 27, 391–400. 10.1523/jneurosci.3709-06.2007 17215400 PMC6672079

[B33] Partridge J. G. Janssen M. J. Chou D. Y. Abe K. Zukowska Z. Vicini S. (2009). Excitatory and inhibitory synapses in neuropeptide Y—expressing striatal interneurons. J. Neurophysiol. 102, 3038–3045. 10.1152/jn.00272.2009 19759327 PMC2777826

[B34] Pennartz C. M. Berke J. D. Graybiel A. M. Ito R. Lansink C. S. van der Meer M.. (2009). Corticostriatal interactions during learning, memory processing, and decision making. J. Neurosci. 29, 12831–12838. 10.1523/jneurosci.3177-09.2009 19828796 PMC3849625

[B35] Picconi B. Bagetta V. Ghiglieri V. Paillè V. Di Filippo M. Pendolino V.. (2011). Inhibition of phosphodiesterases rescues striatal long-term depression and reduces levodopa-induced dyskinesia. Brain 134, 375–387. 10.1093/brain/awq342 21183486

[B36] Rafalovich I. V. Melendez A. E. Plotkin J. L. Tanimura A. Zhai S. Surmeier D. J. (2015). Interneuronal nitric oxide signaling mediates post-synaptic long-term depression of striatal glutamatergic synapses. Cell Rep. 13, 1336–1342. 10.1016/j.celrep.2015.10.015 26549446 PMC4864038

[B37] Szydlowski S. N. Pollak Dorocic I. Planert H. Carlén M. Meletis K. Silberberg G. (2013). Target selectivity of feedforward inhibition by striatal fast-spiking interneurons. J. Neurosci. 33, 1678–1683. 10.1523/JNEUROSCI.3572-12.2013 23345240 PMC6618742

[B38] Tepper J. M. Bolam J. P. (2004). Functional diversity and specificity of neostriatal interneurons. Curr. Opin. Neurobiol. 14, 685–692. 10.1016/j.conb.2004.10.003 15582369

[B39] Tepper J. M. Tecuapetla F. Koós T. Ibáñez-Sandoval O. (2010). Heterogeneity and diversity of striatal GABAergic interneurons. Front. Neuroanat. 4:150. 10.3389/fnana.2010.00150 21228905 PMC3016690

[B300] Tepper J. M. Wilson C. J. Koós T. (2008). Feedforward and feedback inhibition in neostriatal GABAergic spiny neurons. Brain Res. Rev. 58, 272–281. 10.1016/j.brainresrev.2007.10.008 18054796 PMC2562631

[B40] Threlfell S. Lalic T. Platt N. J. Jennings K. A. Deisseroth K. Cragg S. J. (2012). Striatal dopamine release is triggered by synchronized activity in cholinergic interneurons. Neuron 75, 58–64. 10.1016/j.neuron.2012.04.038 22794260

[B41] West A. R. Tseng K. Y. (2011). Nitric oxide—soluble guanylyl cyclase—cyclic gmp signaling in the striatum: new targets for the treatment of Parkinson’s disease? Front. Syst. Neurosci. 5:55. 10.3389/fnsys.2011.00055 21747761 PMC3129139

[B42] Wilson C. J. (2005). The mechanism of intrinsic amplification of hyperpolarizations and spontaneous bursting in striatal cholinergic interneurons. Neuron 45, 575–585. 10.1016/j.neuron.2004.12.053 15721243

[B43] Yamamoto K. Ebihara K. Koshikawa N. Kobayashi M. (2013). Reciprocal regulation of inhibitory synaptic transmission by nicotinic and muscarinic receptors in rat nucleus accumbens shell. J. Physiol. 591, 5745–5763. 10.1113/jphysiol.2013.258558 24018951 PMC3853507

[B44] Zheng T. Wilson C. J. (2002). Corticostriatal combinatorics: the implications of corticostriatal axonal arborizations. J. Neurophysiol. 87, 1007–1017. 11826064 10.1152/jn.00519.2001

